# Supplementary material for: Molecular subtypes of Adenovirus-associated acute respiratory infection outbreak in children in Northern Vietnam and risk factors of more severe cases
Source: PLoS Negl Trop Dis. 2023 Nov 7;17(11):e0011311. doi: 10.1371/journal.pntd.0011311 (PMC10655982; doi:10.1371/journal.pntd.0011311)
Supplement: S1 Table — (DOCX) [file pntd.0011311.s001.docx]

**Supplementary Table S1. List of reference Human Adenovirus (HAdV) strains used in the manuscript’s *hexon* gene phylogenetic comparisons.**

| **GenBank ID** | **Organism** | **Country of origin** | **Collection date** | **Strain Name** |
| --- | --- | --- | --- | --- |
| KU145046.1 | Human adenovirus B3 | Malaysia | 2011-Apr | HAdV3/41362/MYS/2011 |
| MK913813.1 | Human adenovirus B3 | Argentina | 2008-Oct | BA_279-2008 |
| MK736411.1 | Human adenovirus B3 | China | 2016-Oct | B3/Shenzhen173/CHN/2016 |
| KC570906.1 | Human adenovirus B3 | China | 2003-Jan | N78/TW/03 |
| KR090803.1 | Human adenovirus B3 | China | 2011-Aug | GZ_31_2011 |
| MK847517.1 | Human adenovirus B3 | China | 2019-Aug | Shanghai/3496/3[P3H3F3]/2009 |
| MK736413.1 | Human adenovirus B3 | China | 2019-apr | B3/Shenzhen027/CHN/2017 |
| ON661661.1 | Human adenovirus B3 | England | 2022-May | England\19.4\2022\B3 |
| MN011575.1 | Human adenovirus B7 | China | 2019-Jun | SH6220 |
| MK736434.1 | Human adenovirus B7 | China | 2018-Jul | B7/Shenzhen038/CHN/2018 |
| MT350202.1 | Human adenovirus B7 | China | 2017-May | Adv7/Jiangxi/YQ38-1/2017 |
| KU145058.1 | Human adenovirus B7 | Malaysia | 2012-Nov | HAdV7/52792/MYS/2012 |
| KU145113.1 | Human adenovirus B7 | Malaysia | 2013-Jul | HAdV7/22532/MYS/2013 |
| MN507870.1 | Human adenovirus B7 | China | 2019-sep | HAdV/WH/46/2019 |
| MK883612.1 | Human adenovirus B7 | China | 2019-may | Shanghai/6138/7/2014[P7H7F7] |
| MT019923.1 | Human adenovirus B7 | China | 2020-feb | Gansu2015-089 |
| AB685369.1 | Human adenovirus C2 | Mongolia | 2011 | 11_02194/Mongolia/hexon |
| MK913810.1 | Human adenovirus C2 | Argentina | 2008-Jun | BA_24-2008 |
| KC570896.1 | Human adenovirus C2 | China | 2007-May | N1985/TW/07 |
| KU145029.1 | Human adenovirus C2 | Malaysia | 2011-Feb | HAdV2/17102/MYS/2011 |
| MH322442.1 | Human adenovirus C2 | China | 2018-May | Yunnan-CHN/9148/2013 |
| MH322419.1 | Human adenovirus C2 | China | 2018-May | Xizang-CHN/HFMD-134/2015 |
| KU145024.1 | Human adenovirus C2 | Malaysia | 2015-Nov | HAdV2/01636/MYS/2012 |
| MT364185.1 | Human adenovirus C2 | China | 2020-Apr | JiangXiAds996 |
