# Supplementary material for: Molecular subtypes of Adenovirus-associated acute respiratory infection outbreak in children in Northern Vietnam and risk factors of more severe cases
Source: PLoS Negl Trop Dis. 2023 Nov 7;17(11):e0011311. doi: 10.1371/journal.pntd.0011311 (PMC10655982; doi:10.1371/journal.pntd.0011311)
Supplement: S2 Table — (DOCX) [file pntd.0011311.s002.docx]

**Supplementary Table S2. Microbiology and patient characteristics by Human Adenovirus (HAdV) subtypes.**

| **Characteristics** | **C2 (N=1)** | **B3 (N=81)** | **B7 (N=15)** | **P-value comparison #** |
| --- | --- | --- | --- | --- |
| Viral co-detection$$ | 0 (0.0%) | 17 (21.0%) | 3 (20.0%) | 1.000 |
| Bacterial co-detection @@ | 0 (0.0%) | 16 (19.8%) | 4 (26.7%) | 0.609 |
| Any co-detection $@ | 0 (0.0%) | 29 (35.8%) | 6 (40.0%) | 0.857 |
| Sex (males) | 1 (100.0%) | 45 (56.2%) | 7 (50.0%) | 0.874 |
| Age at hospital visit (year) (mean (95%CI)) | 2.000 (NaN, NaN) | 2.763 (2.426, 3.099) | 2.714 (1.742, 3.686) | 0.779 |
| Body mass index (BMI) (kg/m2) (mean (95%CI)) | 15.951 (NaN, NaN) | 15.335 (14.894, 15.775) | 14.833 (14.088, 15.579) | 0.620 |
| Gestational age (week) (mean (95%CI)) | 38.000 (NaN, NaN) | 37.536 (36.488, 38.583) | 38.000 (37.122, 38.878) | 0.806 |
| Delivery method |  |  |  | 0.299 |
| - Cesarean-section | 0 (0.0%) | 21 (32.8%) | 6 (54.5%) |  |
| - Vaginal | 1 (100.0%) | 43 (67.2%) | 5 (45.5%) |  |
| Breast feeding duration (month) (mean (95%CI)) | 12.000 (NaN, NaN) | 11.827 (10.511, 13.143) | 13.300 (9.410, 17.190) | 0.601 |
| History of COVID-19 infection | 1 (100.0%) | 14 (27.5%) | 3 (30.0%) | 0.472 |
| History of allergy | 0 (0.0%) | 3 (3.9%) | 1 (7.1%) | 0.516 |
| History of immuno-compromise diseases | 0 | 0 | 0 |  |
| History of chronic disease | 0 | 0 | 0 |  |
| History of hospitalization due to respiratory disease | 0 (0.0%) | 5 (6.2%) | 2 (13.3%) | 0.350 |
| Day of disease at hospital visit (mean (95%CI)) | 2.000 (NaN, NaN) | 3.568 (3.115, 4.020) | 4.077 (3.139, 5.015) | 0.198 |
| Cough | 1 (100.0%) | 74 (96.1%) | 13 (92.9%) | 0.516 |
| Fever | 1 (100.0%) | 75 (92.6%) | 12 (80.0%) |  |
| Duration of fever (day) (mean (95%CI)) | 5.000 (NaN, NaN) | 6.125 (5.507, 6.743) | 6.818 (5.352, 8.284) | 0.295 |
| Red eye | 0 (0.0%) | 27 (35.5%) | 3 (23.1%) | 0.687 |
| Loose stool | 0 (0.0%) | 12 (15.8%) | 3 (23.1%) | 0.545 |
| Lymph node enlargement | 0 (0.0%) | 5 (6.8%) | 1 (7.1%) | 1.000 |
| Skin rash | 0 (0.0%) | 1 (1.3%) | 0 (0.0%) | 1.000 |
| Coughing up sputum | 1 (100.0%) | 72 (93.5%) | 13 (92.9%) | 1.000 |
| Ear pain | 0 (0.0%) | 4 (5.7%) | 1 (7.1%) | 1.000 |
| Abdominal pain | 0 (0.0%) | 5 (6.6%) | 2 (16.7%) | 0.302 |
| Poor eating | 0 (0.0%) | 30 (40.0%) | 8 (57.1%) | 0.308 |
| White blood cell (WBC) count (x10^9/L) (mean (95%CI)) | 16.800 (NaN, NaN) | 16.181 (14.555, 17.807) | 9.407 (7.110, 11.704) | < 0.001 |
| Neutrophil count (x10^9/L) (mean (95%CI)) | 9.979 (NaN, NaN) | 10.709 (9.341, 12.077) | 4.427 (3.046, 5.808) | < 0.001 |
| Lymphocyte count (x10^9/L) (mean (95%CI)) | 4.334 (NaN, NaN) | 3.516 (3.070, 3.963) | 3.722 (2.698, 4.745) | 0.698 |
| Hemoglobin (g/L) (mean (95%CI)) | 12.600 (NaN, NaN) | 86.588 (74.612, 98.564) | 86.071 (57.757, 114.386) | 0.641 |
| Platelet (x10^9/L) (mean (95%CI)) | 315.000 (NaN, NaN) | 306.033 (283.055, 329.012) | 249.650 (209.645, 289.655) | 0.075 |
| C reactive Protein (CRP) (mg/dL) (mean (95%CI)) | 54.100 (NaN, NaN) | 41.761 (33.285, 50.236) | 33.192 (11.789, 54.595) | 0.348 |
| Chest X-ray abnormality | 0 | 16 (45.7%) | 6 (75.0%) |  |
| Duration of treatment (day) (mean (95%CI)) | 4.000 (NaN, NaN) | 4.981 (4.405, 5.557) | 5.417 (3.672, 7.161) | 0.541 |
| Oxy supplement | 0 (0.0%) | 3 (3.7%) | 3 (20.0%) | 0.106 |
| Intravenous Immunogloblin (IVIG) infusion for Adenovirus treatment | 0 | 1 (1.7%) | 1 (10.0%) |  |
| Nebulizer | 0 | 9 (15.0%) | 1 (10.0%) |  |
| Corticoid use | 0 | 4 (6.9%) | 1 (10.0%) |  |
| Antibiotic use | 1 (100.0%) | 64 (85.3%) | 13 (92.9%) | 0.725 |
| Clinical outcome |  |  |  |  |
| - Dead | 0 (0.0%) | 0 (0.0%) | 0 (0.0%) |  |
| - Discharged without complication | 1 (100.0%) | 68 (98.6%) | 13 (100.0%) |  |
| - Discharged with complication | 0 (0.0%) | 1 (1.4%) | 0 (0.0%) |  |

*#P-values from Kruskal-Wallis’s test for continuous variables or Fisher’s exact test for categorical variables.*

*$$Co-detection with ≥1 other viruses in PCR panel (HEV: human enterovirus, hMPV: human metapneumovirus, PIV1-4: parainfluenza virus 1-4) or Influenza virus type A or type B using rapid antigen test*

*@@Co-detection with ≥1 bacteria in nasopharyngeal fluid culture or PCR panel of 7 respiratory bacteria (Haemophilus influenzae (HI), Streptococcus pneumoniae (SP), Staphylococcus aureus (SA), Moraxella catarrhalis (MC), Mycoplasma pneumoniae (MP), Chlamydophila pneumoniae (CP), Legionella pneumophila (LP)) or Mycoplasma antibody test.*

*$@Any co-detection of ≥1 bacteria or viruses mentioned above.*
