# Supplementary material for: Molecular subtypes of Adenovirus-associated acute respiratory infection outbreak in children in Northern Vietnam and risk factors of more severe cases
Source: PLoS Negl Trop Dis. 2023 Nov 7;17(11):e0011311. doi: 10.1371/journal.pntd.0011311 (PMC10655982; doi:10.1371/journal.pntd.0011311)
Supplement: S1 Protocol — (PDF) [file pntd.0011311.s005.pdf]

Protocol: Molecular typing of Adenovirus by Sanger Sequencing

1. Reagent requires:

   1. AdhexF1 (5′- TICTTTGACATICGIGGIGTICTIGA-3′)
   2. AdhexR1 (5′- CTGTCIACIGCCTGRTTCCACA-3′)
   3. AdhexF2 (5′- GGYCCYAGYTTYAARCCCTAYTC-3′)
   4. AdhexR2 (5′- GGTTCTGTC ICCCAGAGARTCIAGCA-3′)
   5. GoTaq Green Master Mix 2X
   6. Nuclease free water
   7. Agarose Powder
   8. TBE 10X buffer
   9. DNA 1kb Ladder
   10. Red safe TM Nucleic Acid Staining Solution 20000X
   11. BigDye Terminator v3.1 Cycle Sequencing
   12. BigDye X Terminator purification
   13. POP-7 Polymer
   14. Cathode Buffer Container
   15. Anode Buffer Container
   16. Hi-DiTM Formamide
   17. Ethanol 70 %

2. Isolate viral DNA from nasopharyngeal samples

3. Prepare outer PCR using primer pair AdhexF1 and AdhexR1:

	A	B	C
1	Reagent name	Final Concentration	Volume (ul)
2	dH20		8,5
3	GoTaq Green Master Mix 2X	1X	12,5
4	AdhexF1 (10µM)	0.4uM	1,0
5	AdhexR1 (10µM)	0.4uM	1,0

4. Add 2ul of DNA template (20-125ng/ul)

5. PCR thermo cycle:

	A	B	C	D
1	Temp	Time	Cycle	
2	95°C	3 min	1	
3	95°C	30 sec	11	
4	55°C	45 sec		Decrease 1 degree every one cycle
5	72°C	1 min		
6	95°C		20	
7	45°C	45 sec		
8	72°C	1 min		
9	72°C	5 min	1	
10	4°C	∞	1	

6. Check PCR products using electrophoresis on 1-1.5% agarose DNA gel

7. Dilute outer PCR product 1:5-1:15 with dH2O

8. Prepare inner PCR using primer pair AdhexF2 and AdhexR2:

	A	B	C
1	Reagent name	Final Concentratio n	Volume (ul)
2	dH20		9,5
3	GoTaq Green Master Mix 2X	1X	12,5
4	AdhexF2 (10µM)	0.4uM	1,0
5	AdhexR2 (10µM)	0.4uM	1,0

9. Add 1ul of diluted PCR product from step 7

10. PCR thermo cycle:

	A	B	C	D
1	Temp	Time	Cycle	
2	95°C	3 min	1	
3	95°C	30 sec	11	
4	55°C	45 sec		Decrease 1 degree every one cycle
5	72°C	1 min		
6	95°C		20	
7	45°C	45 sec		
8	72°C	1 min		
9	72°C	5 min	1	
10	4°C	∞	1	

11. Check PCR products using electrophoresis on 1-1.5% agarose DNA gel

12. Dilute outer PCR product 1:15 with dH2O

13. Prepare cycle sequencing master mix:

	A	B
1	Reagent	Volume
2	BigDye Terminator	0.5 µl
3	5x Sequencing Buffer	2 µl
4	dH2O	4.65 µl
5	AdhexR2 or AdhexF2 (10 uM)	0.85 µl

14. Cycle sequencing Thermocycler:

	A	B	C
1	96°C	2 min	1 cycle
2	96°C	10 sec	25 cycles
3	50°C	10 sec	
4	60°C	2 min	
5	4°C	∞	1 cycle

15. Purify Cycle sequencing product using Bigdye X Terminator :

	A	B
1	Reagent	Volume
2	SAM solution	45 µl
3	BigDye X-Terminator	10 µl
4	Sample	10 µl

Vortex reactions for 30 minutes and centrifuge at 1000g in 2 minutes

16. Use 15ul of supernatant for Sanger Sequencing on the Applied Biosystems 3500 Dx Genetic Analyzer
