# Supplementary material for: Molecular subtypes of Adenovirus-associated acute respiratory infection outbreak in children in Northern Vietnam and risk factors of more severe cases
Source: PLoS Negl Trop Dis. 2023 Nov 7;17(11):e0011311. doi: 10.1371/journal.pntd.0011311 (PMC10655982; doi:10.1371/journal.pntd.0011311)
Supplement: Attachment — Submitted filename: renamed_8a1d4.docx [file pntd.0011311.s006.docx]

The study stratified and grouped individuals based on the severity of infection and adenovirus genotypes during a local adenovirus outbreak. It combined clinical major symptoms and auxiliary examination results, particularly analyzing co-infection with other pathogens. The research topic and innovation are acceptable, but there are still many shortcomings.

1. The title of the article gives the impression of analyzing the differences in pathogenic characteristics based on different adenovirus genotypes. However, the focus of the text is not on this aspect; it only briefly compares several factors between genotypes after constructing an evolutionary tree.
2. According to clinical reasoning, only patients with more severe conditions would undergo various techniques and multiple-angle screenings for various infectious pathogens. Therefore, it is likely that mild patients did not receive the same level of testing. This leads to the phenomenon of co-infection only appearing in severe patients. If the author did not correct for this confounding factor, the study design and results were undoubtedly meaningless..
3. If the author has already excluded co-infection in mild patients, I would like to ask how the colonization of pathogens was defined and differentiated in the study.
4. How were the potentially risky factors selected in the univariate and multivariate analyses? I understand that they may have come from the statistical analysis in Table 1, but the included factors are not equivalent.
5. The production of figures and tables in the text is relatively rough. Many factors in Table 1 lack units, such as "Age at hospital visit," and my understanding is that its unit should be "days/365". “WBC” also lacks units, which is inappropriate. The comparison between groups in each figure lacks p-values, making it difficult to understand.
6. The discussion is not sufficiently in-depth and mostly repeats the results.
