# Supplementary material for: Molecular subtypes of Adenovirus-associated acute respiratory infection outbreak in children in Northern Vietnam and risk factors of more severe cases
Source: PLoS Negl Trop Dis. 2023 Nov 7;17(11):e0011311. doi: 10.1371/journal.pntd.0011311 (PMC10655982; doi:10.1371/journal.pntd.0011311)
Supplement: Attachment — Submitted filename: Response to Reviewers comments.docx [file pntd.0011311.s007.docx]

**RESPONSE TO REVIEWS**

**Reviewers’ comments**
**Reviewer #1:**

General comments:
Human adenoviruses (HAdVs) are a group of viruses that can cause various illnesses in humans, including respiratory, gastrointestinal, and ocular infections. There are over 50 known serotypes of adenoviruses that can cause human infections. Some serotypes, such as Adenovirus serotypes 3, 4, and 7, are known to be more commonly associated with respiratory tract infections in children. Different serotypes can have varying levels of pathogenicity and clinical manifestations.
This study aimed to investigate the molecular types of HAdVs outbreak and attempted to determine their associations with clinical manifestations of children with respiratory infections in Northern Vietnam in 2022.

Several flaws as outlined below:
1.      Abstract. Methods. It is suggested to described that all the enrolled patients were presented with acute lower respiratory tract infections. Besides, according to the text, the samples used for sequencing included nasopharyngeal aspirates and nasal swabs.

2.      Abstract. Results. It is suggested to describe the total number of samples sent for pathogen detection. Among these samples, 97 was positive for HAdV and proceeded for further sequencing.

3.      Methods. Diagnostic criteria of pneumonia are quite loose. 

4.      Methods. Statistical analysis. (Page 9, Line 162) The Kruskal-Wallis test is designed to compare the medians of three or more independent groups. In this study, the characteristics were compared between two groups (e.g. with and without pneumonia). It is more appropriate to use the Mann-Whitney U test rather than the Kruskal-Wallis test. In addition, Kruskal-Wallis test was misspelled.

5.      Methods/Results. The major drawback of this study is the detection method. Detection of respiratory virus by PCR assay could only indicate the presence of DNA of virus and could not provide sufficient information about infectivity or acute infection. Such carryover detection of nucleic acid of inactivated viruses in certain species could result in an over-weighted effect on epidemiological analysis.

6.      Results. Another major flaw is the definition of co-infection. The results of the microbial agents identified in this study should be co-detection instead of co-infection. Particularly the diagnosis of Mycoplasma pneumoniae infection was based on the antibody test.

7.      Results. The description of the Table 1 is hard to understand. Several characteristics had mysterious denominators. It is suggested to check it carefully.

8.      Results. Table 1. It is suggested to show the neutrophil and lymphocyte counts instead of %. In addition, the unit of platelet count and CRP should be provided.

9.      Results. Figure 1 and 2 are unnecessary. All the information were shown in the Table 1.

10.     Results. Table 2. The number of patients with influenza shown here is inconsistent with the data described in the text.

11.     Results. Figure 4. The numbers of tested adenovirus 3 and 7 were inconsistent with the data described in the text.

12.     There are several typos in the manuscript. The authors should check it carefully. English editing is also highly recommended.

Response: Thanks for your contributive comments. Below are our responses:

1. The study included 97 patients who had positive HAdV PCR results with Ct<30 (to ensure the success of subsequent sequencing). Clinical data were collected retrospectively for these 97 patients. These patients were then classified into two groups: those who had upper respiratory illnesses only and those who had lower respiratory illnesses (further classified into those with severe pneumonia or without). In brief, this study included pediatric patients with ARI tested positive with HAdV in PCR and with a Ct<30. We have clarified this in the abstract method section and in the manuscript text (section Sample and Data Collection). Due to word count restriction, it is difficult to include detailed description in the abstract.

2. We have added the summary of samples as suggested in result section “HAdV assays and HAdV subtypes” (as word count restriction does not allow us to put this in the abstract).

3. We have revised the definition of severe pneumonia in the manuscript text section “Sample and Data Collection” as: “Patients were classified as having severe pneumonia if having lower respiratory manifestation such as rales, rhonchi, wheezing and/or lung lesions as indicated by chest x-ray and respiratory distress requiring oxygen supplementation or respiratory support”.

4. The Kruskal-Wallis test may be used for the comparison between two or more groups while Mann-Whitney U test may be used for the comparison between two groups. For two groups, the results of these two tests would be similar. We replaced Kruskal-Wallis test by Mann-Whitney U test in manuscript text as suggested.

5. We agree that a patient with a HAdV positive PCR result does not mean that the patient has HAdV infection. This study was conducted during an outbreak of acute respiratory infection (ARI) reportedly associated with HAdV. The nasal swab and/or nasopharyngeal samples of patients with ARI symptoms were tested for HAdV by PCR panel. Those with HAdV positive PCR results with Ct<30 were included in our study. Blood serologic test for HAdV was not done. The included patients had relatively strong HAdV positive PCR result and relevant clinical respiratory manifestations but of course it is difficult to confirm that their ARI was due to HAdV infection. As such, we now use the term “detection” instead of “infection” and “HAdV-associated ARI outbreak” instead of “respiratory HAdV infection outbreak” in our manuscript to address this concern.

6. We agree and have replaced “co-infection” by “co-detection” in our manuscript.

7. We have added some clarification for some characteristics and footnote in Table 1. The calculated percentage might vary for different variables because missing values varied for different variables (the denominators might vary for different variables due to missing values).

8. We have adjusted the results in Table 1 accordingly.

9. We have removed Figure 1 and Figure 2 of the previous manuscript version.

10, 11, 12. We have fixed the errors/typos and revised the manuscript text accordingly.

**Reviewer #2:**

The study stratified and grouped individuals based on the severity of infection and adenovirus genotypes during a local adenovirus outbreak. It combined clinical major symptoms and auxiliary examination results, particularly analyzing co-infection with other pathogens. The research topic and innovation are acceptable, but there are still many shortcomings.
First, it was not stated whether patients with a single adenovirus positive infection had the same severity of illness as those with multiple pathogens, since only patients with more severe disease were more likely to be tested for multiple pathogens. Second, the chart is too rough, some of the conventional notes are not in place, making it difficult to understand. In addition, the discussion section retells a lot of results, which should further deepen the significance of the research.

Response: Thanks for your contributive comment. PCR panel detecting 7 viruses including HAdV was performed for all patients included in this study (not just for more severe patients). As such the significant association of co-detection of other viruses together with HAdV included in the PCR panel and disease severity is meaningful. This is also in line with some published reports that co-detection of HAdV together with other respiratory viruses increased disease severity as compared to detection of HAdV alone. This is now mentioned and cited in the Discussion. We have added Table 2 summarizing microbial assays done for the patients included in this study. We have also revised the tables-figures and added some further discussion in the Discussion section.

**Reviewer #3:**
The analysis presented matches the analysis plan and the results are clearly presented as well as discussed in the context of the aim. The numbers stated in Table 1 for the HAdV-3 and 7 does not match what is stated in the discussion. This could just be a minor typing or calculation error.

The conclusions are supported by the data presented and limitation of the analysis are clearly described but recommendation for further research are not discussed.  Discussion on how the data can be helpful to advance our understanding on adenovirus infections and associated risks for severe disease are clearly stated. It is clear to the reader that HAdV3 and 7 were the predominant types in the outbreak of respiratory HAdV infection among children in Northern Vietnam and that co-infection of HAdV with other respiratory pathogens is a significant risk factor for severe respiratory infection

Response: Thanks for your contributive comment. We have added recommendation for further research in the discussion section and have fixed the typing error.

In this paper, Nguyen et al report about molecular typing of human adenovirus associated with an outbreak of respiratory infection among children in Northern Vietnam and risk factors for more severe cases.

Comments for minor revision

1.      In the introduction, first paragraph line 48 -50, the authors should mention more than 100 serotypes and refer to the homepage of the adenovirus working group (HAdV Working Group ([gmu.edu](http://gmu.edu)) ). They should also mention that HAdV can cause severe or fatal cases even death in both immunocompetent and immunocompromised individuals.

2.      As the study involves identifying HAdVs types, please describe how typing of HAdVs is performed in the Introduction to give the reader a clear understanding.

3.      The references for HAdV outbreaks with respiratory illnesses, conjunctivitis, and gastrointestinal disorders should be include in line 51 -52 instead of bunching all the references in line 54

4.      Please use the same abbreviation for the same types of HAdV e.g either use HAdV-2  or HAdV-C2 throughout the manuscript not both. See line 54 -58

5.      Line 72 – there should not be a  full stop  between  “ acute respiratory distress . which required intensive treatment and prolonged hospitalisation) replace with a comma

6.      Since HAdV is a DNA virus, the viral extract would be DNA not RNA.  Would recommend replacing “ partly used to extract RNA by QIAamp Viral RNA Kit “ with  subjected to nucleic acid extraction using the QIAamp Viral RNA Kits within 24 hours after collection (line 96-97). Also  “Extracted RNA samples “ should be replaced with Extracted nucleic acids were tested for HAdV and 6 other respiratory pathogens.

7.      In the method section line 103, can you please briefly state how the HAdV molecular typing was done i.e was it based on the hexon or fibre or penton base gene or both hexon and fibre genes. If hexon gene what region i.e 1-6 or 1-7 hypervariable region

8.      Lines 124 -128 – HadV should be HAdV. Please correct throughout the manuscript

9.      Lines 140  - replace “only sample with a detectable appropriate PCR product from nested-PCR “ with  only samples showing amplicons with the expected band size from nested-PCR ……

10.     What was the classification for the history of severe respiratory disease? This is not mentioned in the methods.

11.      Line 201 –  Bacterial_coinfection not “Vacterial_coinfection”. Also either use coinfection or co-infection throughout the manuscript not both

12.      Although typing of HAdV based on hexon 1-6  may be sufficient for rapid identification of HAdV types during an outbreak or for routine large-scale molecular epidemiological monitoring,  it is not enough to identify HAdV types according to the sequence of hypervariable regions 1-6 of the hexon gene as HAdVs are prone to recombination. please discuss the limitation of using only the hexon for HAdV typing.

13.      There is inconsistency in the number of samples that were identified as HAdV-3 and 7. The number in Table 1 for HAdVs identified as type 3 and 7 (81 and 15 samples respectively)  does not match what is stated in the results section line 310 (82 samples for type 3 and 14 samples for type 7) and also in the discussion line 343.  Line 310 “ Ninety-seven (98.98%) HAdV samples were genotyped successfully”, shouldn’t this be 100% of the samples instead of 98.98% as stated?

Response: Thanks for your contributive comments. Our responses are below.

1. We have added the suggested content in the first paragraph of the Introduction section.

2,3,4,5,6,7,8,9,11,13: We have corrected these issues in the manuscript text.

10. We have replaced “history of severe respiratory disease” by “history of prior hospitalization due to respiratory disease” for better clarification.

12. We have added some discussion about the limitation of using only the hexon for HAdV typing in the “Discussion” section.

**Reviewer #4:**

- Was the presence of RSV in the clinical specimens investigated?  Is there a reason why this prevalent respiratory pathogen  was not included?
-Was fever part of the diagnostic criteria?
-The acronym for human adenovirus is HAdV. Please correct throughout the manuscript.
-On line 124 the correct term to describe the coding region targeted for amplification and molecular typing is HEXON, not exon.
-What is the limit of detection of the diagnostic PCR used to detect HAdV in the study?
-On line 152 please provide the URL for NCBI.
-The spelling of GenBank needs to be corrected.
-On line 154 a reference needs to be cited for ClustalW.
-What parameter is the cutoff value of <80% referring to?
-On line 173, please correct the text to read qPCR.

Response: Thanks for your contributive comments.

We agree that it is a limitation of this study that Respiratory syncytial virus (RSV), a common respiratory virus in children with acute respiratory infection (ARI), was not tested. The study samples were selected based on HAdV positive result of the PCR panel as the initial aim of our study is to perform molecular subtyping of HAdV due to the rapidly rising number of Adenovirus- associated ARI cases in Northern Vietnam from the beginning of Autumn 2022 (news link: <https://outbreaknewstoday.com/vietnam-adenovirus-cases-have-increased-dramatically-at-the-national-childrens-hospital-22079/#:~:text=Specifically%2C%20as%20of%20September%2012,Six%20deaths%20have%20been%20recorded>). In Vinmec General Hospital, we used commercial kit Allplex™ Respiratory Panel 2 to detect Adenovirus and the other 6 ARI viruses which unfortunately did not include RSV. Therefore, some respiratory viruses including RSV were left out in this study due to this technical limitation. We have added the discussion about this limitation in the Discussion section of our manuscript.

Fever was among the clinical features evaluated in this study but was not among diagnostic criteria.

The limit of detection of the diagnostic PCR used to detect HAdV in the study was quantification cycle threshold (Ct) value < 38. Samples with Ct <38 for HAdV were considered HAdV-positive. We described this in the section “Sample and Data Collection” and we have also added clarification to the Result section “HAdV assays and HAdV subtypes” of the revised version of the manuscript.

The cutoff value of <80% refers to the bootstrap support threshold used in constructing the phylogenetic tree. When constructing a phylogenetic tree using MEGA software's bootstrap method, the included bootstrap values indicate the confidence level of each branch and are available in the output file. We only consider branches with bootstrap values lower than 80% for our phylogenetic analysis. Therefore, we provide additional explanation that the cutoff value of <80% refers to the bootstrap support threshold used in constructing the phylogenetic tree (section “Data analysis”, sub-section “Sequence data and phylogenetic analysis”). It means that branches with a bootstrap support value below 80% were not included in the final tree. This choice was made to focus on more strongly supported relationships while acknowledging uncertainty in some regions of the dataset.

We have revised the manuscript accordingly to address the above comments.

-Was a documented previous COVID-19 infection a risk factor for HAdV-associated disease severity?
-On line 226, what does "day of illness" mean?
-Please use the symbol "°C to describe temperature.
-On line 311, please correct the virus type to read B7.
-Were the hexon amplicon sequences deposited in GenBank?
-The legend of figure 3 needs revision.
 This study provides molecular epidemiology data for HAdV respiratory infections in Vietnam for the first time since the early 2000. The study provides important-albeit modest- information regarding prevalent HAdV types circulating in the area.

The manuscript would benefit significantly from  a revision of the use of language by a native speaker of English

The INTRODUCTION section needs a brief description of the current classification of adenoviruses and a consistent nomenclature to describe HADV species and types.

The REFERENCES provided by the authors starting on line 400 are incomplete.

The wording of the title of this manuscript needs revision in many sections.

Response: Thanks for your contributive comments. Previous COVID-19 infection was documented in 18 (29.0%) of all patients included in this study. Clinical and laboratory data were collected retrospectively so missing data is one of the limitations of our study and we acknowledged this in the Discussion section.

“Day of illness” or “Day of disease” is the duration time (in days) from the date of disease onset to the date of hospital visit. We have clarified this in the result section “Patient characteristics and clinical features”.

We have submitted the representative hexon sequences of HAdV-B3, C2 and B7 to GenBank and the submission ID are 2732596, 2732634 and 2732640 respectively. After the submission is approved, we will add the GenBank ID of these sequences into Supplementary Table S1.

We have revised the manuscript text, tables and figures accordingly to address the above comments.

**Other comments** (attached file):

The study stratified and grouped individuals based on the severity of infection and adenovirus genotypes during a local adenovirus outbreak. It combined clinical major symptoms and auxiliary examination results, particularly analyzing co-infection with other pathogens. The research topic and innovation are acceptable, but there are still many shortcomings.

1. The title of the article gives the impression of analyzing the differences in pathogenic characteristics based on different adenovirus genotypes. However, the focus of the text is not on this aspect; it only briefly compares several factors between genotypes after constructing an evolutionary tree.

2. According to clinical reasoning, only patients with more severe conditions would undergo various techniques and multiple-angle screenings for various infectious pathogens. Therefore, it is likely that mild patients did not receive the same level of testing. This leads to the phenomenon of co-infection only appearing in severe patients. If the author did not correct for this confounding factor, the study design and results were undoubtedly meaningless..

3. If the author has already excluded co-infection in mild patients, I would like to ask how the colonization of pathogens was defined and differentiated in the study.

4. How were the potentially risky factors selected in the univariate and multivariate analyses? I understand that they may have come from the statistical analysis in Table 1, but the included factors are not equivalent.

5. The production of figures and tables in the text is relatively rough. Many factors in Table 1 lack units, such as "Age at hospital visit," and my understanding is that its unit should be "days/365". “WBC” also lacks units, which is inappropriate. The comparison between groups in each figure lacks p-values, making it difficult to understand.

6. The discussion is not sufficiently in-depth and mostly repeats the results.

Response: Thanks for your contributive comments. Below are out responses.

1. We did analyze the differences in pathogenic characteristics based on different adenovirus subtypes. However, there were no notable difference between subtypes except for the higher WBC and Neutrophil count in the group with HAdV type B3. Therefore, we only summarized the main characteristics in Figure 2 and in the manuscript text. We have now added the full results in Supplementary Table S2. We have also moved the HAdV phylogenetic tree Figure to Figure 1 and moved the description of HAdV subtyping result to the first result section “HAdV assays and HAdV subtypes” in this revised version of the manuscript.

2. We fully agree with this comment. Actually, the PCR panel detecting 7 viruses including HAdV was performed for all patients included in the study. The participants were selected retrospectively based on the HAdV positive result of the PCR panel. Then clinical manifestations and the results of other viruses on the same PCR panel were examined retrospectively. The co-detection with other viruses detected by PCR panel (which was done for all participants) was significantly associated with lower respiratory illnesses and severe pneumonia even after adjusting for other factors. This indicates that the finding is robust and meaningful statistically and clinically. We have added a table (Table 2) summarizing microbial assays done and description in the section “Other microbiology assays and co-detection of other respiratory viruses or bacteria” of this revised version of the manuscript.

3. Clinical and other microbial data were obtained retrospectively for the cases with positive HAdV in PCR panel. The PCR panel detects 7 viruses including HAdV and 6 other respiratory pathogens (human enterovirus (HEV), human metapneumovirus (hMPV) and parainfluenza virus 1-4 (PIV1-4). The PCR panel was done for all patients with acute respiratory infection (ARI) suggesting HAdV infection included in this study. Influenza viruses were detected by antigen rapid test. Mycoplasma pneumoniae was detected by blood serology test. Bacteria were identified mostly by nasal-pharyngeal swab culture. We have added a summary of microbial assays done for all patients, those with lower respiratory illnesses and those with severe pneumonia in Table 2 and corresponding description in the “Patient characteristics and clinical features” section. Blood serologic tests were not done for the viruses or bacteria detected from nasal swabs or nasopharyngeal aspirates. As such, we acknowledge that it is not possible to clearly differentiate the clinical symptoms of HAdV and of other respiratory pathogens in pediatric patients with ARI. It is also not possible to clearly differentiate between colonization and infection or between co-infection and co-detection. Therefore, we now use the term “detection” and “co-detection” in the manuscript instead of “infection” and “co-infection”. We now also use the term “HAdV-associated ARI outbreak” instead of “acute respiratory HAdV infection outbreak” in our manuscript.

4. Potential risk factors included in the multivariate model were selected if they were clinically meaningful or statistically significant from the univariate model. Univariate model was done for all factors in Table 1. We have revised the manuscript text to clarify this (section “Analysis of HAdV types and clinical contexts”)

5. We have revised the tables and figures accordingly (adding unit, p-value, etc)
6. We have revised the discussion section to add more discussion of the results.

We would like to expressly thank the Editorial Board and the reviewers for all constructive, thoughtful, and especially detailed comments for our manuscript. We feel fortunate to have learned so many helpful tips that will serve us well for this paper and also into the future.
