# Supplementary material for: Molecular subtypes of Adenovirus-associated acute respiratory infection outbreak in children in Northern Vietnam and risk factors of more severe cases
Source: PLoS Negl Trop Dis. 2023 Nov 7;17(11):e0011311. doi: 10.1371/journal.pntd.0011311 (PMC10655982; doi:10.1371/journal.pntd.0011311)
Supplement: Attachment — Submitted filename: 20231010_VMEC_response to reviews _rv2.docx [file pntd.0011311.s008.docx]

**RESPONSE TO REVIEWS**

**Reviewers’ comments**
**Reviewer #1:** Several bacterial species names are misspelled in the text and footnote of tables. For example, Staphylococcus aureus, Legionella pneumophila, etc. The authors should check it carefully throughout the manuscript.

**Response**: Thank you very much for pointing out the spelling errors regarding bacterial species names. We have checked and corrected those errors throughout the manuscript.

Figure Files:

We have used PACE (<https://pacev2.apexcovantage.com>) to ensure that figures meet PLOS requirements.

Data Requirements:

The numerical data used in all figures are included in supporting information file “231010_VMEC_datafigure12”.

We are unable to submit the hexon sequences into Genbank database because the achieved sequences do not cover the whole hexon region to be sufficient for annotation. Instead, we provide all sequencing results used for Figure 1 in supporting information file “231010_VMEC_datafigure12” (sheet Figure 1).

Reproducibility:

We have submitted our laboratory protocol name “Molecular typing of Adenovirus by Sanger Sequencing” in protocols.io. The DOI is: dx.doi.org/10.17504/protocols.io.ewov1qzxogr2/v1. We also would like to send the copy of this protocol in the supporting information file “231010_VMEC_protocolio”.

References:

We have revised the references which were truncated erroneously due to software bugs.
